# Role of sex hormones and the vaginal microbiome in susceptibility and mucosal immunity to HIV-1 in the female genital tract

**DOI:** 10.1186/s12981-017-0169-4

**Published:** 2017-09-12

**Authors:** Danielle Vitali, Jocelyn M. Wessels, Charu Kaushic

**Affiliations:** 1 Department of Pathology and Molecular Medicine, McMaster Immunology Research Centre, McMaster University, MDCL Room 4014, 1280 Main Street West, Hamilton, ON L8S 4K1 Canada; 2 Department of Pathology and Molecular Medicine, McMaster University, Hamilton, ON Canada

**Keywords:** HIV-1 susceptibility, Mucosal immunity, Female genital tract, Sex hormones, Microbiome

## Abstract

While the prevalence of Human immunodeficiency virus-1 (HIV-1) infection has stabilized globally, it continues to be the leading cause of death among women of reproductive age. The majority of new infections are transmitted heterosexually, and women have consistently been found to be more susceptible to HIV-1 infection during heterosexual intercourse compared to men. This emphasizes the need for a deeper understanding of how the microenvironment in the female genital tract (FGT) could influence HIV-1 acquisition. This short review focuses on our current understanding of the interplay between estrogen, progesterone, and the cervicovaginal microbiome and their immunomodulatory effects on the FGT. The role of hormonal contraceptives and bacterial vaginosis on tissue inflammation, T cell immunity and HIV-1 susceptibility is discussed. Taken together, this review provides valuable information for the future development of multi-purpose interventions to prevent HIV-1 infection in women.

## Background

Human immunodeficiency virus-1 (HIV-1) infection remains one of the most serious health challenges in the world, and the fastest growing phase of this pandemic is currently by heterosexual transmission in women [1]. Adolescent girls and young women are at particularly high risk of HIV-1 infection, accounting for 20% of new HIV-1 infections globally. The gender imbalance is even more pronounced in geographical areas with higher HIV-1 prevalence, such as sub-Saharan Africa, where women account for almost 56% of the total number of people living with HIV-1 [1]. Although the female genital mucosa is a major portal for entry of HIV-1 into the body, responsible for initiation of 40% of global HIV-1 infections, the acute events that follow HIV-1 exposure in the female genital tract (FGT) still remain unclear [2].

The mucosal immune system of the female reproductive tract is one of the first lines of defense against incoming pathogens, but has also evolved to support an immunologically distinct fetus, a unique feature at this mucosal site. Female sex hormones estradiol and progesterone have marked immunoregulatory influence, coordinating immune cell phenotype and function and helping to regulate pregnancy and the menstrual cycle (reviewed in [3]). The FGT is compartmentalized between the lower and upper genital tract. The lower FGT consists of the vagina and the ectocervix, and is protected by a stratified squamous epithelium, which relies on the presence of multiple cell layers to provide a protective barrier. Conversely, the upper FGT consists of the endocervix, uterus, fallopian tubes and ovaries, and is lined by a monolayer of columnar epithelium. To support reproductive success, a pattern has evolved in which tissue specific aspects of innate, humoral and cellular immunity are either enhanced or suppressed in the upper and lower FGT, in coordination with hormonal fluctuations throughout the menstrual cycle. For example, during the progesterone-high secretory phase of the cycle, uterine cytotoxic T lymphocyte (CTL) activity and natural killer (NK) cell cytotoxic activity are suppressed whereas innate components are enhanced. While the resulting immune changes optimize the environment for successful embryonic implantation in the upper FGT, they may also increase the risk of acquiring sexually transmitted infections (STIs) at this point in the menstrual cycle, known as a “window of vulnerability” (reviewed in [3, 4]).

Lying superficial to the epithelial cells of the vaginal tract is the microbiome, which exists in a symbiotic relationship with the female host. The current concept of a “healthy” vaginal microbiome includes a low-diversity, *Lactobacillus* rich environment. Four species of *Lactobacilli* (*L. crispatus*, *L. gasseri*, *L. iners*, and *L. jensenii*) are known to be the most common dominant species in the vaginal microbiome of 80–90% of Caucasian and Asian women, and 60% of Black and Hispanic women (reviewed in [5]). Although relatively stable throughout the rapid hormonal shifts of the menstrual cycle, the major hormonal shifts that occur at puberty and menopause significantly change the composition of the vaginal microbiome from mainly anaerobic bacteria to a vaginal microbiome dominated by species of *Lactobacilli*. Estrogen has been implicated in this shift, and a clear relationship between estradiol and colonization with *Lactobacilli* can be found in post-menopausal women on hormone replacement therapy, albeit via an imprecisely known mechanism.

*Lactobacilli* contribute to immunity in the FGT by providing non-specific defense against a broad range of pathogens (reviewed in [6]). They produce pH-modulating lactic acid and hydrogen peroxide, anti-microbial bacteriocins, and form adherent colonies on epithelial cells or cause co-aggregation between bacterial species, providing a physical/neutralizing barrier to protect against other harmful bacterial strains and pathogens. The ability of the host to tolerate Lactobacilli but protect against pathogenic bacteria relies on the bi-directional relationship that exists between the mucosal immune system and the microbiome [7, 8]. Culture-based studies have shown that commensal bacteria do not elicit cytokine release from vaginal epithelial cells, while pathogenic strains induce a strong pro-inflammatory response, likely via activation of epithelial toll-like receptors (TLRs) and interaction with cervical antigen presenting cells. The ability of vaginal bacteria to manipulate mucosal immunity and barrier properties has the potential to result in enhanced susceptibility to infection, particularly during a disturbance in the vaginal microbiome.

Despite the fact that the majority of HIV-1 infections in women occur as a result of heterosexual intercourse with an infected male partner, the precise mechanisms of sexual transmission in the FGT remain elusive. Heterosexual transmission models suggest that HIV-1 in the male ejaculate must first overcome numerous innate and adaptive immune factors in the vaginal lumen [9]. If successful, the virus will traverse through the genital epithelium via tears in the squamous epithelium or transcytosis across the single cell layer of the endocervix, ultimately infecting underlying CD4^+^ target cells in the submucosa. Here, the virus establishes a small founder population of productive infection that then expands systemically, likely via an influx of newly recruited target cells caused by an upregulation of chemokines [10]. Interestingly, studies show that in the majority of infected individuals only 1–3 virus variants are responsible for establishing productive infection in the newly infected partner [11]. There remains to be a clear consensus regarding (a) the primary location of HIV-1 entry in the FGT and (b) the etiology of epithelial penetration by HIV-1.

Although HIV-1 transmission can happen anywhere along the FGT, the columnar epithelium of the endocervix and transformation zone have been proposed as the favored sites for HIV-1 transmission, largely because it is composed of a single layer of cells with a thickness of only 10–30 μm, placing the virus in closer proximity to intraepithelial and submucosal target cells [9]. This is also the most immunologically active site in the FGT with relatively greater abundance of HIV-1 target cells: CD4^+^ T cells and macrophages [12]. Nevertheless, the squamous epithelium of the lower FGT is the primary area that comes in contact with seminal fluid containing infectious virus and comprises the majority of the exposed surface area of the FGT that would arguably present greater access sites for HIV-1 entry, particularly when breaches occur in the epithelium [13]. Together, these results suggest that genital tract acquisition of HIV-1 may take place at a variety of different tissues, and elucidating the mechanisms associated with the early events of HIV-1 infection in both the lower and upper FGT will prove valuable to the design of effective prophylactic therapeutics.

What is exceedingly clear is that increased mucosal inflammation enhances the rate of sexual transmission of HIV-1 in the FGT [10]. Recently, Masson et al. observed a threefold increased risk of HIV-1 infection in South African women who had elevated levels of at least five mucosal pro-inflammatory cytokines, including IL-8, IL-1β, IL-1α and TNF-α [14]. Our lab has shed light on the pathophysiological mechanism by which inflammation induced upon viral exposure can facilitate viral transmission [15, 16]. We showed that HIV-1 envelope protein gp120 interacts with TLR2 and TLR4 on the genital epithelium, resulting in the downstream induction of pro-inflammatory cytokines, such as TNF-α, impairment of barrier function and significant viral translocation across the epithelium. However, the effects of inflammation on HIV-1 transmission extend beyond barrier disruption. Arnold et al. found increased frequencies of CD4^+^ T cells in the endocervix of women with pro-inflammatory cytokine profiles, which is relevant as HIV-1 preferentially infects CD4^+^ T cells, particularly T helper type 17 (Th17) CD4^+^ T cells and activated CD4^+^ T cells expressing α_4_β_7_ or α_4_β_1_ [17]. Indeed, an innate and adaptive inflammatory cascade in response to viral exposure in the FGT is necessary for the recruitment of target cells to the portal of entry and the establishment of a productive, systemic infection.

The vaginal microbiome can also influence susceptibility to HIV-1 via its intimate interaction with mucosal immunity in the vaginal tract. Bacterial vaginosis (BV) is a symptomatic clinical condition diagnosed using the Nugent score or Amsel criteria, characterized by a polymicrobial vaginal microbiome and overgrowth of anaerobes. Although it has been consistently linked to increased risk of acquiring STIs including HIV-1 (reviewed in [5], [18]), it is becoming increasingly clear that bacterial diversity, even in the absence of BV might also confer greater susceptibility to disease [19, 20]. Pro-inflammatory cytokine levels in the vaginal fluid of women with BV are often upregulated, suggesting that BV and perhaps microbial diversity in the absence of BV may be capable of inducing sub-clinical inflammatory responses in the vaginal mucosa which could alter disease susceptibility. Diversity of the cervicovaginal microbiome has also been shown to be associated with changes in the vaginal proteome which might serve to physically disrupt the mucosal barrier [21]. Thus, BV, and likely microbial diversity are able to modify risk to STIs via their interaction with mucosal immunity within the FGT and modification of its protective epithelial barrier.

There is also emerging evidence that commonly used hormonal contraceptives may increase the risk of HIV-1 acquisition and transmission. Injectable progestins, including depot medroxyprogesterone acetate (DMPA) and norethisterone enanthate (NET-EN), are the favored form of contraception used by approximately 8 million women in sub-Saharan Africa [22]. Although it remains controversial in the literature, numerous observational studies have identified DMPA as a significant risk factor for the acquisition of HIV-1. In a recent prospective cohort study, the HIV-1 incidence in South African women using DMPA and NET-EN was 2.93 times higher than the incidence in those not using long-term contraception [23]. While the biological mechanism remains unclear, women using injectable progestin-only contraceptives had 3.92 times the frequency of cervical CCR5^+^ CD4^+^ T cells compared to women not using long-term contraception who were in the naturally progesterone-high luteal phase of the menstrual cycle. Disruption of the epithelial barrier, promotion of HIV-1 replication and transcytosis, suppression of innate and adaptive immune responses and changes in the vaginal microbiome are all additional proposed mechanisms through which DMPA may increase the risk of HIV-1 transmission (reviewed in [24]). To date, a few studies have attempted to elucidate the effect of hormonal contraceptives on the vaginal microbiome [21, 25, 26]. Some studies find subtle shifts and changes in women on hormonal contraceptives including enhancement of *Lactobacillus* species or reduction in total bacterial load, *Gardnerella vaginalis* and *Lactobacilli* [25–28]. Others have demonstrated that the incidence of BV is decreased in women using both oral contraceptives and DMPA [29–31]. While this may suggest that alterations in the vaginal microbiome is an unlikely explanation for the increased risk of HIV-1 acquisition in DMPA users, the effect of DMPA use on the vaginal microbiome has not been fully elucidated, and a comprehensive and well controlled assessment of the effect of hormonal contraceptives on the vaginal microbiome as it relates to HIV-1 susceptibility is still lacking. Given the accumulating evidence regarding safety of DMPA in women at high risk for HIV-1 acquisition, the World Health Organization has recently revised its guidelines to state that women can use DMPA but should be advised about the increased risk of HIV-1 acquisition and take steps to minimize their risk.

Thus a deeper understanding of the genital microenvironment, including cross talk between microbiome, female sex hormones, naturally-occurring and contraceptive containing synthetics, and the mucosal immune system could inform the use and development of (a) safer hormonal contraceptives and (b) sex-based vaccines against HIV-1. Future interventions for the prevention of HIV-1 infection could be gender-specific and multipurpose, combining vaccines with local probiotics or anti-inflammatory compounds to counter immune activation in the FGT. Curcumin [32] and glycerol monolaurate [33, 34] are examples of potential measures to counter immune activation in the FGT and may prove effective by decreasing target cell recruitment and the expansion of infected founder populations at the portal of entry. By increase vaginal *Lactobacillus* species and limiting tissue inflammation at the site of infection, these potential therapeutics in combination with an HIV-1 vaccine may enhance protection against the acquisition or spread of HIV-1 infection.

## Conclusion

**Fig. 1:**
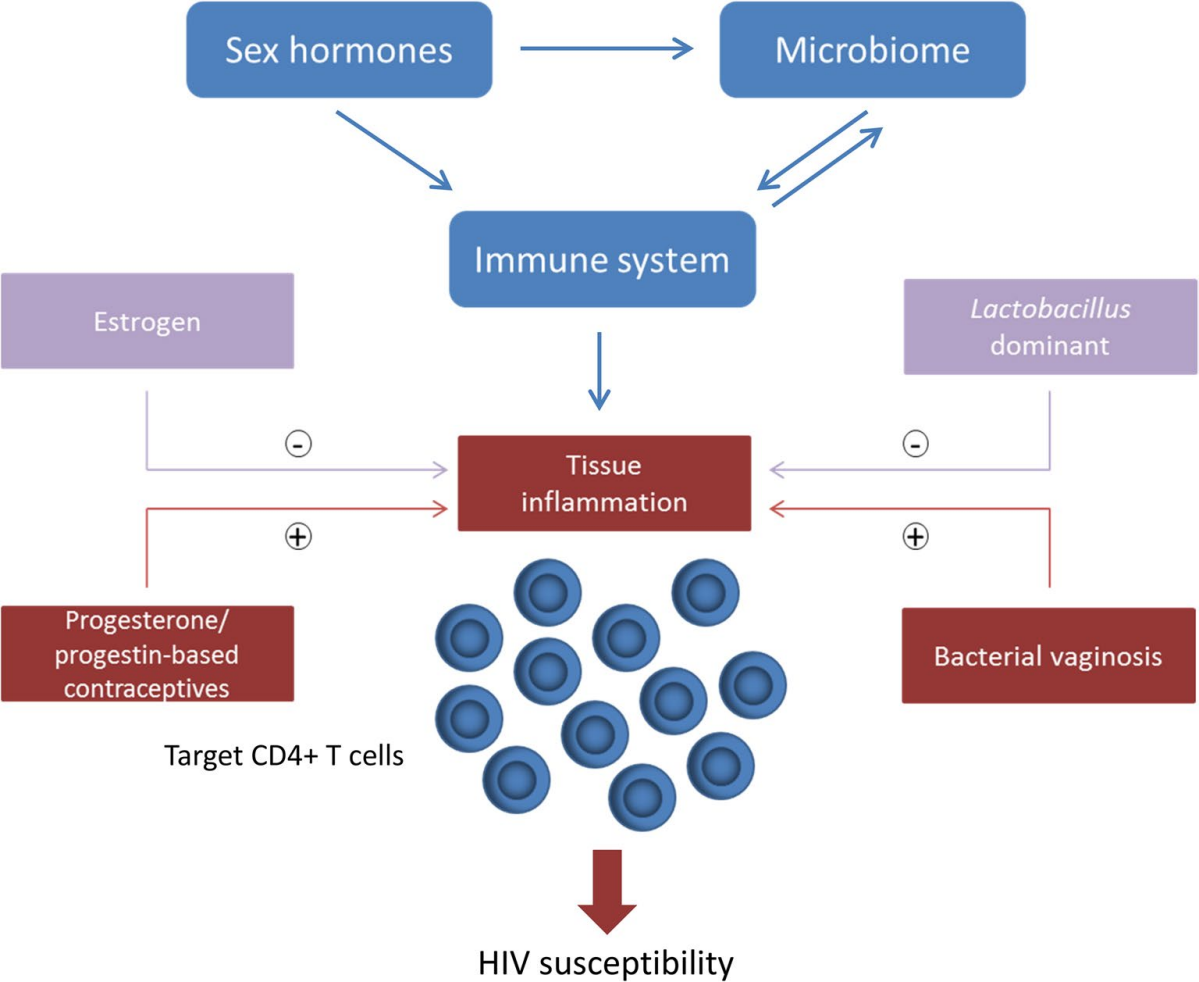
The sex hormone-microbiome-immune system axis in the female genital tract (FGT). The hormonal milieu, consisting of estradiol and progesterone, regulates the vaginal microbiome and both these factors participate in cross-talk with the immune system in the FGT (*blue*), determining the level of innate inflammation in the genital tissue. Higher estrogen levels correlate with a vaginal microbiome dominated by *Lactobacillus* spp. which can decrease genital inflammation and reduce HIV-1 susceptibility (*pink*). Alternatively, the use of progestin-based contraceptives or the presence of BV can initiate an inflammatory cytokine microenvironment that attracts T cells and induce their activation (*red*). Elevated levels of CD4^+^ CCR5^+^ activated T cells in the tissue as a result of an inflammatory genital profile increases the risk of HIV-1 acquisition in women

The FGT is a key target site for HIV-1 transmission in women, and the outcome of exposure to HIV-1 is likely determined by a number of factors that influence this mucosal microenvironment. It is unique among mucosal sites, challenged with the need to enable successful reproduction as well as mediate protection against sexually transmitted infections, such as HIV-1. Estrogen, progesterone, hormonal contraceptives and the vaginal microbiome are all factors within the microenvironment that participate in cross-talk with the immune system (Fig. 1). The net outcome of these interactions that results in an inflammatory microenvironment could be favorable for HIV-1 infection and replication by attracting target cells, which will subsequently become infected and further propagate the infection. The biological mechanisms underpinning the association between DMPA use or BV and increased HIV-1 susceptibility, although not conclusively established, likely rely on their ability to enhance mucosal inflammation and target cell recruitment within the female genital tract. A better understanding of the interplay between sex hormones, the vaginal microbiome and the immune system could inform strategies for development of multi-purpose interventions to prevent HIV-1 infection in women.

## Abbreviations

FGT: female genital tract
CTL: cytotoxic T lymphocyte
STIs: sexually transmitted infections
TLRs: toll-like receptors
Th17: T helper type 17
BV: bacterial vaginosis
DMPA: depo medroxyprogesterone acetate
NET-EN: norethisterone enanthate
